# How the Covid-19 pandemic affected relationships, connectedness, and suicidality among asexual spectrum adults

**DOI:** 10.5249/jivr.v17i1.1906

**Published:** 2025-01

**Authors:** Brian N. Fink

**Affiliations:** ^*a*^ University of Toledo Health Science Campus, Department of Population Health, 3000 Arlington Ave., MS# 1027, Toledo, Ohio 43614, United States of America.

**Keywords:** Relationships, Connectedness, Pandemic, Suicide, Asexual

## Abstract

**Background:**

The impact of COVID-19 on personal and social relationships was considerable. Specifically, social distancing, meant to prevent disease spread, may have adversely impacted personal and social relationships. Suicidal ideation has been found to be more likely due to various health factors, including the isolation that was a result of the pandemic itself. While sex and gender minorities are often studied as a group, it is unknown whether the pandemic adversely affected the relationships and connectedness to others among individuals identifying on the asexual spectrum. Further, how might these effects have increased or decreased their risk of suicidality?

**Methods:**

Data from the 2022 Ace Community Survey was analyzed. Questions specifically pertaining to COVID-19 and the effects it had on relationship desire, as well as connectedness to others were assessed, along with suicidality, among adults identifying on the asexual spectrum.

**Results:**

Those with an increased desire for queerplatonic relationships had a greater risk of both thoughts and attempts of suicide. Asexual individuals who felt less connected to parents, other family, and roommates had a greater risk of suicidal thoughts. However, those who felt more connected with mental health workers and healthcare professionals had an increased risk of thoughts and attempts of suicide. The more educated an asexual spectrum adult, the less likely they were to be suicidal.

**Conclusions:**

Relationships and connectivity among asexual spectrum adults were adversely affected by the pandemic, resulting in increased risk of suicidality. Creating ways to foster relationships among this population are important in advance of the next pandemic to ensure greater health and well-being.

## Introduction

What it means to be asexual is an absence of sexual attraction to others.^1-3^ However, it is a misnomer to consider the asexual population as a monolithic group that is not interested in sex. The asexual identity spectrum includes those who identify as demisexual, considered to be an individual feeling sexually attracted to another once they have developed strong connections on an emotional level; and gray-asexual, considered to be a person someone that may experience attraction on a sexual level, but does not expect the relationship to be based on sexual attraction. ^2^ Given the asexual spectrum population has diversity just like those who comprise the non-asexual population, it would be interesting to study how the recent outbreak of COVID-19 impacted their relationships, connectedness, and suicidality. 

The pandemic adversely affected social relationships through increased separation from other people, including family, friends, co-workers, teachers, and healthcare providers.^4-5^ Social distancing restricted the number and range of people with whom physical affection was possible.^6^ Specifically, distancing increased distress in relationships among couples, and reduced relationship quality among family members.^4^ School closures reduced in-person social interaction among students and increased their experiences of loneliness and psychological distress. ^7^ Among older adults, the pandemic increased loneliness and isolation, sparking the need for strategies that foster connectedness over time.^8^ The adverse effects of social distancing include altered relationships between people, including rude behavior, lack of boundaries, isolation, and decreased effort in romantic relationships and friendships. ^9-11^ The instability of emotional relationships has been associated with an increased risk of suicide among adolescents. ^12^ It is possible the pandemic adversely affected the relationships and connectivity with others among asexual spectrum individuals. 

This mandated isolation may have also increased the risk of suicide.^13^ Meta-analysis data collected prior to the pandemic, indicate those who are minorities by sex and gender (SGM) are more likely to commit suicide and suffer self-injury compared to heterosexual individuals.^14-15^ Similarly, pandemic data indicated a greater risk of suicidality among adults.^16^ In 2003, the Severe Acute Respiratory Syndrome (SARS) pandemic increased the likelihood of social disengagement and suicide, despite the fact that social distancing was nowhere as pervasive and strict. ^17^ SGM populations have reported a greater risk of anxiety, depression, suicidal ideation, and reduced social and family support compared to others.^18-20^

Among 12,449 people from around the world, who identified as asexual, nearly one-third reported thinking about suicide, 10.6% had planned suicide, and 2.7% had attempted suicide in the prior year.^21^ Similarly, the prevalence of suicidality has been observed to be greater among those who identify as asexual compared to those who do not.^10^ However, neither of these studies assessed the potential impact of COVID-19. Therefore, it would be helpful to study how the pandemic may have adversely impacted individuals identifying on the asexual spectrum. 

A plethora of research has indicated suicidal ideation increased during the COVID-19 pandemic.^22^ This increased risk may be due to less social interaction, isolation, and increased anxiety.^23-24^ A Kaiser Family Foundation survey found that nearly half of American adults (45%) stated the pandemic had a negative effect on their mental health, primarily from worrying and stressing about COVID-19.^25^ Even among medical workers treating patients during the pandemic, loneliness, exhaustion, little to no support from others, and difficulty staying mentally healthy increased their risk of suicide.^26^ Though suicidal ideation may not result in attempted or completed suicide, the psychological and social effects of COVID-19 may be risk factors for suicide.^26^ Being away from others can manifest itself in mental health disorders and suicidal thoughts and actions.^17,27-30^ Émile Durkheim stress staying connected with others to promote mental health.^27^

Relationships are a vital support system for SGM individuals, especially among those who socially distanced with their families.^31^ This diverse population has been negatively impacted, particularly socially, during COVID-19.^32^ Physical, mental, and financial health and well-being; as well as social connectivity have been worsened more compared to heterosexual and cisgender sexual minority individuals, resulting from the pandemic.^33-34^ (The majority of cisgender, sexual minority individuals were less connected socially as a result of the pandemic.^33^ During the pandemic, young SGM adults had a greater risk of depression, anxiety, and feeling isolated from their friend.^35-40^

Despite the ever-increasing research, there have been no published studies specifically focused the potential negative impacts of the pandemic on the relationships and suicidality of those who are part of the asexual spectrum community. Notably, the term ‘asexual’ is not explicitly named within the LGBTQ+ acronym. Rather, it can be interpreted to be within the + portion. It is, however, stated when a different acronym, specifically naming those who identify as intersex or asexual (LGBTQIA+), is utilized. Those part of the asexual community are, in essence, a minority within the SGM. Therefore, the adverse social and personal relationship impacts related to pandemic policies deserve attention. Further, the diversity of the asexual population may reveal the negative pandemic impacts will affect them similarly to the non-asexual population. 

This research aims to study how the pandemic impacted the desire for relationships, social connectedness, and suicide among adults identifying on the asexual spectrum. It is expected the experiences of asexual individuals are like those of sexual individuals. They simply need to be studied as their own population. The hypotheses of this work are the following: 1) those who feel less connected to others will be at a greater likelihood to engage in suicidal behavior than those who felt no change or more connected; and 2) those who have decreased desire for various relationships will be more likely to engage in suicidal ideation than those who have no change or increased desire.

## Methods 

This non-human subject research project work earned approval from the university Institutional Review Board (IRB) (approval number 301998-UT). The Ace Community Survey is conducted annually and is provided in an online format. It is designed and disseminated by the Ace Community Survey Team.^41^ In the 2022 Ace Community Survey, there was a section of questions included about the impact of the pandemic on others. The exact text to begin this section was the following: “The following section contains questions about the impact of COVID-19. Are you willing to answer these questions?” These questions inquired specifically about their feelings of connectedness to various groups and desire for various types of relationships. 

In this section of twelve questions, respondents were asked to pick their one level of connectedness (less connected, no change, more connected) to each of the following groups: partners; parents; other family members; roommates or non-family household members; asexual or aromantic communities; LGBTQIA+ friends; non-LGBTQIA+ friends; classmates; teachers and school staff; co-workers; mental health professionals; and medical professionals. In the analysis, for each question, more connected was compared to those who responded less connected or no change.

In this section of nine questions, respondents were asked to pick their one level of desire (decreased, no change, increased, unsure) for the following: romantic relationship, sexual relationship, queerplatonic relationship, friendship, cohabitation, domestic partnership or civil union, marriage, familial relationship, and other.

The dependent variables were those dealing with suicide in the past year. Suicidal ideation was assessed in the survey through the following questions: “During the past twelve months, did you ever seriously consider suicide?” and “How many times did you actually attempt suicide?”41 Those who indicated they had attempted suicide at least once were considered to have ever attempted suicide, and thus, compared to those who never attempted suicide.

Statistical Package for the Social Sciences (SPSS) version 29.0 (Armonk, NY: IBM Corp.) was used to analyze the data. Study population demographics were tabulated with frequencies. Multiple logistic regression modeled the effects of the pandemic relationship issues and suicidal ideation, adjusted for the demographic variables. Chi-square analysis was used to determine if pandemic-impacted connectedness and suicidal ideation were associated.

## Results

A total of 8,824 adults on the asexual spectrum completed the 2022 Ace Community Survey (Table 1). Of this study sample, 3,533 (40.0%) identified as female, 876 (10.0%) identified as male, and 4,407 (49.9%) identified as non-binary. The sexual orientation to which most identified with was asexual (63.9%), followed by demisexual (11.6%), gray-asexual (10.5%), and questioning (7.9%). Ages of the individuals ranged from 18 to 86 years with a mean age of 26.44 years and a standard deviation of 7.76 years. 

**Table 1:** Demographics Characteristics of Study Population (n = 8,824)

Demographic Characteristics	N (%)/ Mean (SD)
**Age**	26.44 (7.76)
**Gender**	
Woman or female	3,533 (40.0%)
Man or male	876 (10.0%)
Non-binary	4,407 (49.9%)
Missing	8 (0.1%)
**Asexual Identity**	
Asexual	5,638 (63.9%)
Gray-asexual	930 (10.5%)
Demisexual	1,019 (11.6%)
Questioning	698 (7.9%)
Missing	539 (6.1%)
**Education**	
Less than Secondary	281 (3.2%)
Complete Secondary Education	1,205 (13.7%)
Some College	2,782 (31.5%)
Completed Vocational or Trade Program	286 (3.2%)
Completed Associate’s or Bachelor’s	2,824 (32.0%)
Completed Graduate Degree	1,414 (16.0%)
Missing	32 (0.4%)
**Employment Status**	
Employed, paid, part/full	4,430 (50.8%)
Not employed and looking and/or on disability	956 (11.0%)
Not employed and not looking	392 (4.5%)
Student	2,707 (31.1%)
Caregiver/Parent	51 (0.6%)
Retired	23 (0.3%)
Volunteer	103 (1.2%)
Missing	53 (0.6%)

After adjusting for age, asexual spectrum identification, employment status, education, and gender, those who reported an increased desire for a queerplatonic relationship were more likely to consider suicide (OR = 1.14, 95% CI: 1.17, 1.79) and attempt suicide (OR = 1.71, 95% CI: 1.09, 2.68) (Table 2). There was also an increased risk of suicidal consideration among those with increased desire for a sexual relationship, though not quite statistically significant (OR = 1.37, 95% CI: 0.97, 1.72), and with increased desire for a domestic partnership or civil union (OR = 1.39, 95% CI; 1.04, 1.86). 

**Table 2:** Adjusted logistic regression modeling of COVID-19-impacted relationship desire on suicidality in the past twelve months

	Suicidal Ideation		Suicide Attempts	
	AOR 95% CI	p	AOR 95% CI	p
**Age **	0.98 (0.96,0.99)	.002	0.97 (0.93,0.99)	.03
**Gender**				
Woman or female	1 [Reference]		1 [Reference]	
Man or male	1.15 (0.87,1.52)	.33	1.94 (1.12,3.37)	.02
Non-binary	1.50 (1.25,1.80)	<.001	1.38 (0.91,1.08)	.12
**Asexual Identity**				
Asexual	1 [Reference]		1 [Reference]	
Gray-asexual	1.11 (0.87,1.44)	.40	1.04 (0.58,1.87)	.90
Demisexual	1.39 (1.10,1.75)	.01	2.23 (1.44,3.47)	<.001
Questioning	1.45 (1.07,1.97)	.02	1.54 (0.84,2.82)	.17
**Education **				
Less than Secondary	1 [Reference]		1 [Reference]	
Complete Secondary Education	0.70 (0.44,1.14)	.15	0.76 (0.34,1.69)	.50
Some College	0.58 (0.37,0.92)	.02	0.61 (0.28,1.32)	.21
Completed Vocational or Trade Program	0.68 (0.37,1.23)	.20	0.57 (0.19,1.78)	.33
Completed Associate’s or Bachelor’s	0.37 (0.23,0.60)	<.001	0.41 (0.18,0.95)	.03
Completed Graduate Degree	0.27 (0.16,0.46)	<.001	0.12 (0.04,0.41)	<.001
**Employment**				
Employed, paid, part/full	1 [Reference]		1 [Reference]	
Not employed and looking and/or on disability	1.93 (1.49,2.51)	<.001	1.32 (0.77,2.27)	.31
Not employed and not looking	1.02 (0.63,1.64)	.93	1.21 (0.52,2.82)	.65
Student	0.97 (0.78,1.20)	.76	0.89 (0.58,1.39)	.62
Caregiver/Parent	0.99 (0.33,2.90)	.99	4.04 (1.10,14.88)	.04
Retired	N/A	N/A	N/A	N/A
Volunteer	2.03 (1.16, 3.57)	.01	.85 (0.20,3.62)	.83
**Romantic Relationship**				
No change	1 [Reference]		1 [Reference]	
Unsure	0.93 (0,66,1.29)	.65	1.11 (0.57,2.16)	.76
Increased	1.10 (0.87,1.38)	.41	0.89 (0.54,1.47)	.65
Decreased	0.89 (0.58,1.36)	.59	1.67 (0.79,3.56)	.18
**Sexual Relationship**				
No change	1 [Reference]		1 [Reference]	
Unsure	1.33 (1.02,1.73)	.04	0.84 (0.48,1.47)	.54
Increased	1.37 (0.97,1.92)	.07	1.13 (0.57,2.21)	.73
Decreased	0.84 (0.52,1.38)	.50	0.60 (0.21,1.69)	.33
**Queerplatonic Relationship**				
No change	1 [Reference]		1 [Reference]	
Unsure	1.65 (1.00,2.74)	.05	1.26 (0.46,3.41)	.66
Increased	1.44 (1.17,1.79)	<.001	1.71 (1.09,2.68)	.02
Decreased	1.02 (0.70,1.50)	.91	0.81 (0.35,1.87)	.62
**Friendship**				
No change	1 [Reference]		1 [Reference]	
Unsure	1.23 (0.83,1.83)	.30	0.91 (0.39,2.10)	.82
Increased	0.84 (0.68,1.03)	.10	0.97 (0.62,1.53)	.90
Decreased	0.87 (0.43,1.77)	.71	1.73 (0.55,5.49)	.35
**Cohabitation**				
No change	1 [Reference]		1 [Reference]	
Unsure	0.93 (0.69,1.25)	.61	1.34 (0.75,2.39)	.32
Increased	0.84 (0.67,1.06)	.13	0.67 (0.40,1.12)	.13
Decreased	0.68 (0.44,1.07)	.09	0.80 (0.34,1.87)	.60
**Domestic Partnership/Civil Union**				
No change	1 [Reference]		1 [Reference]	
Unsure	1.34 (0.87,2.07)	.19	2.01 (0.89,4.53)	.09
Increased	1.39 (1.04,1.86)	.03	1.28 (0.69,2.37)	.44
Decreased	1.53 (0.99,2.37)	.06	1.06 (0.44,2.57)	.90
**Marriage**				
No change	1 [Reference]		1 [Reference]	
Unsure	1.07 (0.77,1.47)	.69	0.83 (0.43,1.60)	.58
Increased	0.99 (0.71,1.38)	.96	0.88 (0.43,1.82)	.73
Decreased	0.80 (0.47,1.37)	.42	0.80 (0.27,2.37)	.69
**Familial**				
No change	1 [Reference]		1 [Reference]	
Unsure	1.56 (1.16,2.11)	.01	1.37 (0.78,2.40)	.28
Increased	1.01 (0.80,1.27)	.92	0.76 (0.46,1.28)	.31
Decreased	1.04 (0.63,1.73)	.87	0.86 (0.31,2.40)	.78
**Other**				
No change	1 [Reference]		1 [Reference]	
Unsure	0.85 (0.48,1.50)	.57	0.99 (0.36,2.76)	.98
Increased	0.94 (0.60,1.48)	.79	2.43 (1.13,5.23)	.02
Decreased	1.28 (0.97,1.70)	.08	1.81 (1.05.3.12)	.03

Note. AOR is the abbreviation for adjusted odds ratio Adjusted for age, gender, asexual identity, education, and employment. CI is the abbreviation for confidence interval.

## Discussion

Asexual spectrum adults who felt more connected with mental health and medical professionals, due to the pandemic, were more likely to engage in suicidal consideration and attempts(Table 3 ). However, individuals that felt less connected with parents, other family, and roommates were statistically more likely to consider suicide. Those who felt less connected with roommates were also more likely to attempt suicide.

**Table 3:** Connectedness and Suicidal Ideation in the Past Twelve Months

	Consider Suicide		P	Attempt Suicide		P
	Yes N (%)	No N (%)		Yes N (%)	No N (%)	
**Partners **						
More connected	191 (22.9%)	644	.14	34 (3.7%)	894	.95
Less or no change	1,121 (25.3%)	3,318		181 (3.6%)	4,821	
**Parents**						
More connected	375 (20.9%)	1,421	.03	64 (3.2%)	1,950	.71
Less or no change	1,259 (23.4%)	4,120		183 (3.0%)	5,888	
**Other Family **						
More connected	191 (18.1%)	866	<.01	33 (2.8%)	1,163	.57
Less or no change	1,448 (23.7%)	4,661		211 (3.1%)	6,667	
**Roommates **						
More connected	171 (20.8%)	652	.01	20 (2.2%)	1,180	.03
Less or no change	1,135 (24.9%)	3,428		184 (3.6%)	4,983	
**Ace/Aro Communities **						
More connected	406 (23.0%)	1,359	.54	64 (3.2%)	1,945	.93
Less or no change	1,095 (23.7%)	3,520		167 (3.2%)	5,009	
**LGBTQIA+ Friends**						
More connected	477 (27.7%)	1,458	.13	82 (3.7%)	2,138	13
Less or no change	1,099 (22.9%)	3,693		162 (3.0%)	5,191	
**Non-LGBTQIA+ Friends**						
More connected	181 (22.8%)	614	.83	33 (3.6%)	876	.40
Less or no change	1,399 (23.1%)	4,665		211 (3.1%)	6,576	
**Classmates**						
More connected	38 (21.6%)	138	.34	7 (3.3%)	205	.88
Less or no change	1,352 (24.7%)	4,112		216 (3.5%)	5,963	
**Teachers and Staff**						
More connected	35 (24.5%)	108	.96	6 (3.4%)	169	.94
Less connected or no change	1,341 (24.7%)	4,095		217 (3.5%)	5,926	
**Coworkers**						
More connected	110 (20.9%)	417	.20	10 (1.7%)	574	.03
Less or no change	1,243 (23.3%)	4,087		200 (3.3%)	5,779	
**Mental Health Workers**						
More connected	294 (29.5%)	702	<.01	54 (4.8%)	1,070	.01
Less or no change	1,180 (24.9%)	3,559		175 (3.3%)	5,162	
**Medical Professionals**						
More connected	145 (33.2%)	292	<.01	31 (6.3%)	459	<.01
Less or no change	1,341 (23.7%)	4,316		200 (3.1%)	6,163	

Note. Chi-square analysis used to compare observed proportions of connectedness between asexual spectrum adults and these groups.

A lack of social integration, support, and connection implies that contacts and relationships are disrupted or non-existent and can result in feeling like one does not belong.^13,27,42-43^ It is plausible that among those with more positive mental health, suicidal ideation would be less likely. In fact, those reporting better mental health have demonstrated less risk of suicidal ideation, even when faced with stressful situations.^44^ Better mental health may be demonstrative of better resilience. In this study, it was observed that those who agreed the Covid-19 pandemic negatively impacted their mental health were statistically more likely to consider suicide, and those who agreed that the pandemic made their living situation more stressful were more likely to consider, plan, and attempt suicide. Results found throughout this research further demonstrate the need for affordable or free mental health service accessibility, particularly during a pandemic. 

Prior to the pandemic, research indicated that a small fraction (5%) of people considered suicide.^45^ These adverse impacts to mental health and well-being have been observed among asexual individuals. Secondary students in New Zealand not merely attracted to the opposite sex had a greater likelihood of visiting a mental health professional and having more difficulty accessing help for emotional concerns.^46^ However, this research did not focus on the asexual spectrum. 

A cross-sectional survey such as the 2022 Ace Community Survey makes it more difficult to assess potential causality between pandemic-impacted factors and suicide. A cohort study would better assess the potential temporality between COVID-19 and suicide. The findings were based on self-report and therefore were subject to recall bias and biases related to social desirability. Though the information collected were provided by online survey, participant ages ranged from 18 to 86 years. Additionally, there is no data source that approaches the sample size of individuals identifying on the asexual spectrum provided by the 2022 Ace Community Survey.

The use of sampling methods that involve the snowball technique and targeting on sites focused on asexual individuals, as well as other social media outlets, helps reach difficult-to-access populations. However, it may diminish the generalizability of conclusions from this work to all asexual individuals.

Queerplatonic relationships consist of dedicated, long-term partnership where the feelings are not romantic, but still strong.^2^ These relationships may be misinterpreted as evidence of heterosexuality if they happen between cross-gender partners, and evidence of homosexuality if they occur between same-gender partners.^2^ Perhaps the increased suicidality among those with an increased desire for a queerplatonic relationship is related to those who feel less connected to other family members and roommates who are also at greater risk of suicidality. A decreased connection of those bonds, resulting from the pandemic, could be playing a role in these phenomena. 

Demisexual adults had a greater likelihood of considering and attempting suicide compared to gray-asexual and asexual adults. As demisexual individuals generally feel sexually attracted once they feeling strongly connected to someone, it should not be surprising that they were more likely to be suicidal in the past year, even after adjustment for multiple variables, including asexual identity.^2^ It is important to understand that desire for sexual relationships do occur among those on the asexual identity spectrum, particularly for those who identify as demisexual. When that desire cannot be met, in part, because of a pandemic where social distancing was enacted to limit the spread of an infection, that suicidal thoughts are possible. 

Relationships were impacted by the COVID-19 pandemic and it stands to reason that those impacts would likely be negative. Pandemic policies undeniably affected relationships and perhaps the most influential of these policies was social distancing. Social distancing inherently is meant to keep people apart to prevent the spread of infectious disease. Thus, social distancing may be associated with people feeling less connected and feeling like they have less social support, and thus increasing their risk for suicidal ideation. 

The adverse pandemic effects on social and personal relationships, as well as mental and physical health, may have increased the likelihood that asexual spectrum individuals would want to seek healthcare. Similarly, those who were already mental and/or medical health patients may have been at a heightened susceptibility to COVID-19 on their health and, therefore, had greater desire to see their healthcare providers.

The results of this work highlight the need for supporting individuals identifying on the asexual spectrum and may help inform tailored mental health and additional methods to help, including culturally-appropriate programs designed to prevent suicide (Lyons et al., 2022). Fostering relationships with social connectedness may help mitigate distress in the face of a pandemic (Jacmin-Park et al., 2022). Relationships inherently involve connection between people. Helping those on the asexual spectrum to maintain and build new relationships is critical to their health and well-being.

**Acknowledgement**

Authorship Confirmation/Contribution Statement: I am the only author. For the CRediT taxonomy, I did all the contributions to this work.
